# Supplementary figures and images for: The transpeptidase PBP2 governs initial localization and activity of the major cell-wall synthesis machinery in E. coli
Source: eLife. 2020 Feb 20;9:e50629. doi: 10.7554/eLife.50629 (PMC7089770; doi:10.7554/eLife.50629)

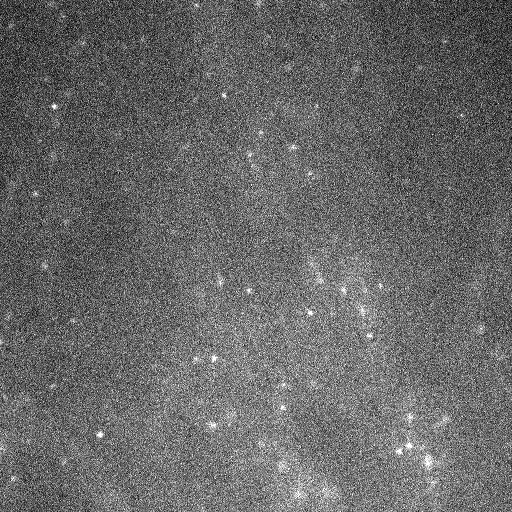

Supplement: Source code 1. [file elife-50629-code1.zip › PAmCherry-PBP2/slow/samplemovie/Pos0/img_000000006_Default1_000.tif]

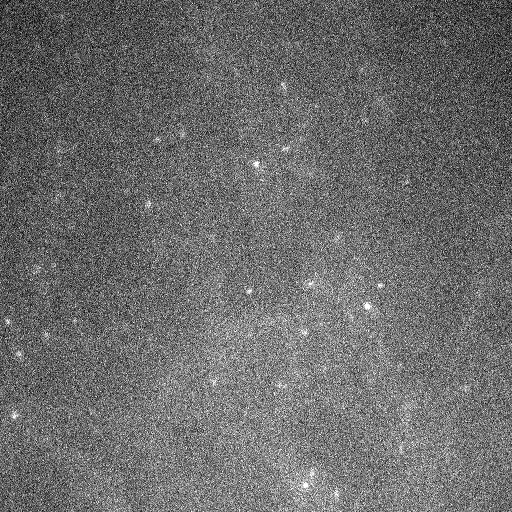

Supplement: Source code 1. [file elife-50629-code1.zip › PAmCherry-PBP2/slow/samplemovie/Pos0/img_000000027_Default1_000.tif]

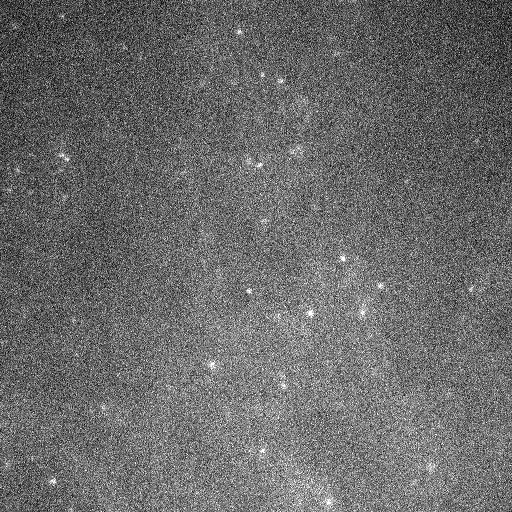

Supplement: Source code 1. [file elife-50629-code1.zip › PAmCherry-PBP2/slow/samplemovie/Pos0/img_000000019_Default1_000.tif]

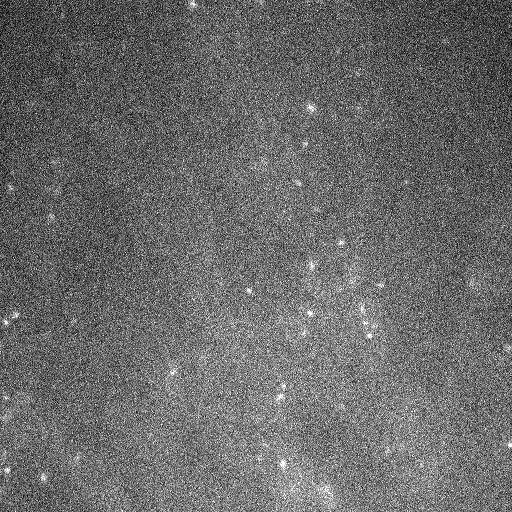

Supplement: Source code 1. [file elife-50629-code1.zip › PAmCherry-PBP2/slow/samplemovie/Pos0/img_000000012_Default1_000.tif]

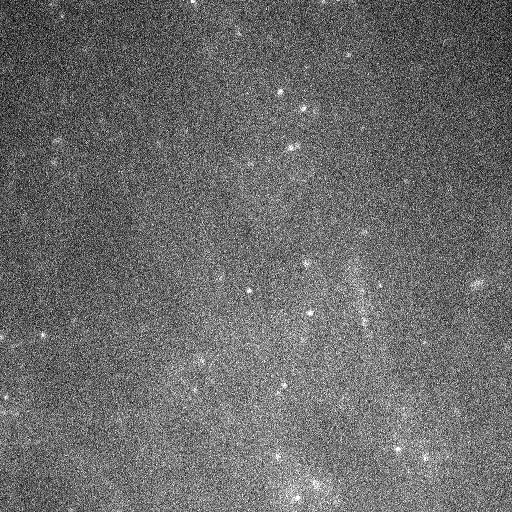

Supplement: Source code 1. [file elife-50629-code1.zip › PAmCherry-PBP2/slow/samplemovie/Pos0/img_000000016_Default1_000.tif]

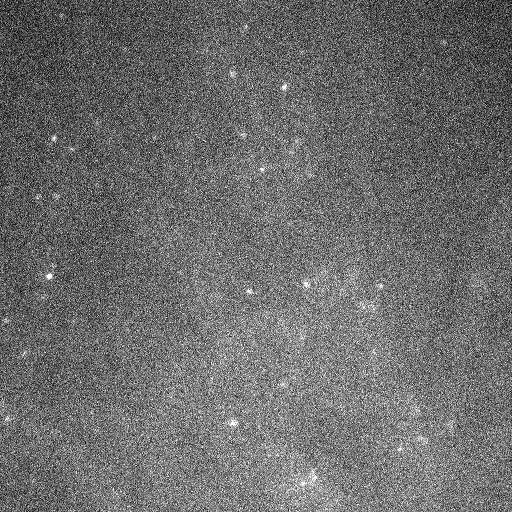

Supplement: Source code 1. [file elife-50629-code1.zip › PAmCherry-PBP2/slow/samplemovie/Pos0/img_000000028_Default1_000.tif]

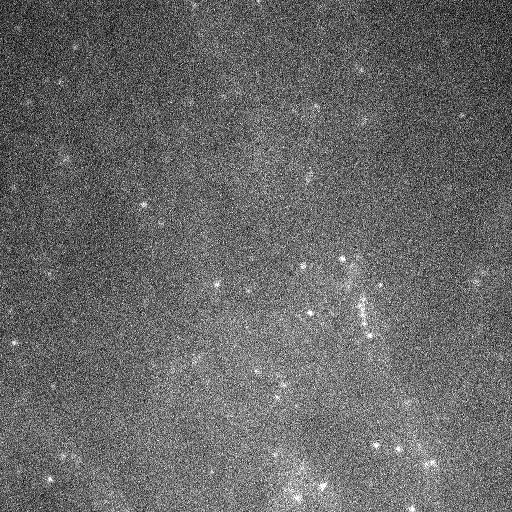

Supplement: Source code 1. [file elife-50629-code1.zip › PAmCherry-PBP2/slow/samplemovie/Pos0/img_000000009_Default1_000.tif]

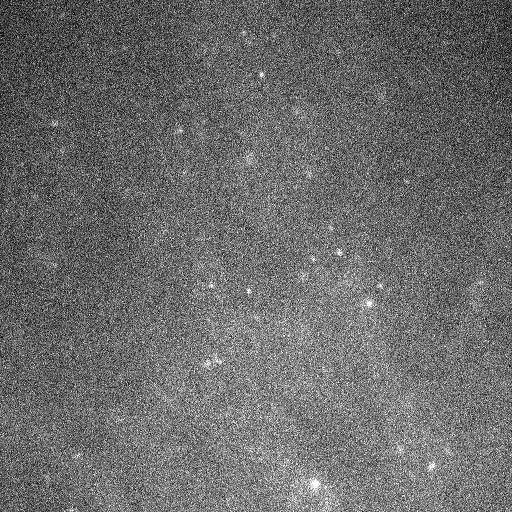

Supplement: Source code 1. [file elife-50629-code1.zip › PAmCherry-PBP2/slow/samplemovie/Pos0/img_000000023_Default1_000.tif]

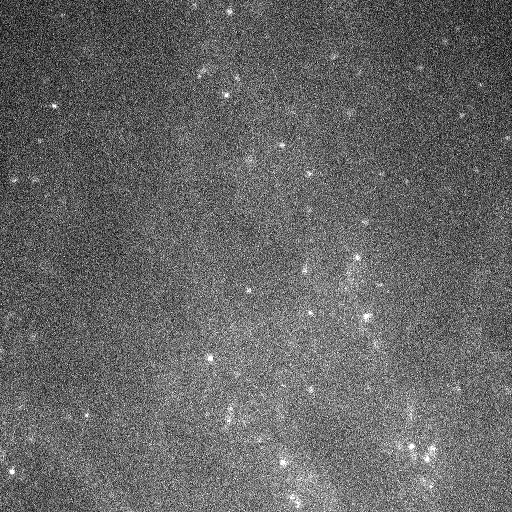

Supplement: Source code 1. [file elife-50629-code1.zip › PAmCherry-PBP2/slow/samplemovie/Pos0/img_000000002_Default1_000.tif]

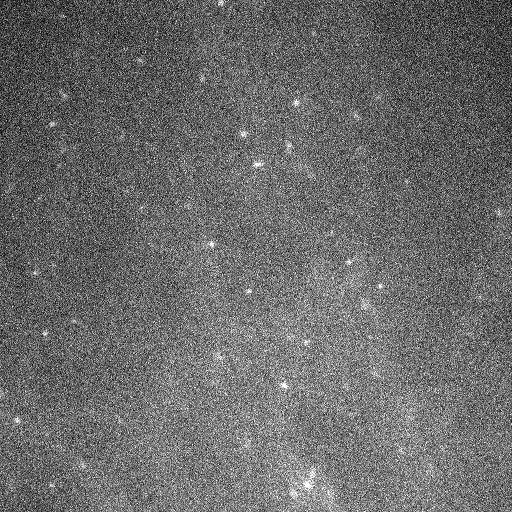

Supplement: Source code 1. [file elife-50629-code1.zip › PAmCherry-PBP2/slow/samplemovie/Pos0/img_000000026_Default1_000.tif]

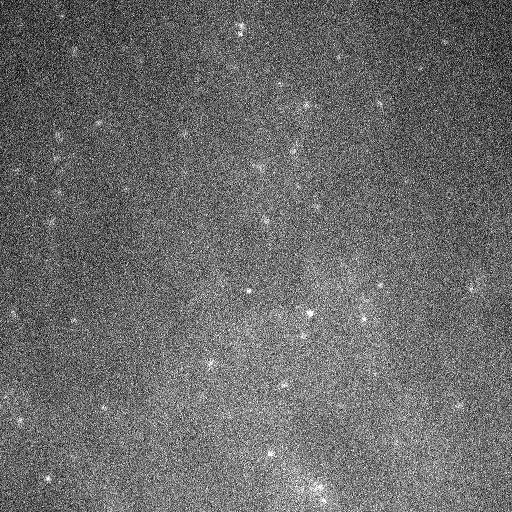

Supplement: Source code 1. [file elife-50629-code1.zip › PAmCherry-PBP2/slow/samplemovie/Pos0/img_000000018_Default1_000.tif]

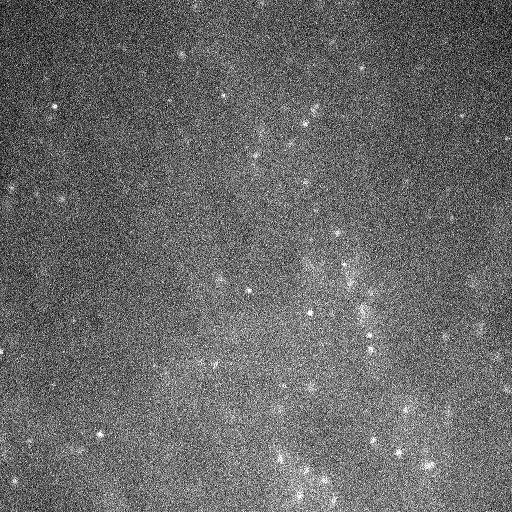

Supplement: Source code 1. [file elife-50629-code1.zip › PAmCherry-PBP2/slow/samplemovie/Pos0/img_000000007_Default1_000.tif]

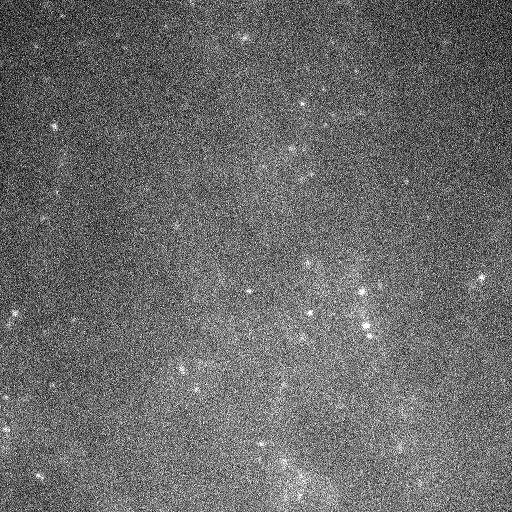

Supplement: Source code 1. [file elife-50629-code1.zip › PAmCherry-PBP2/slow/samplemovie/Pos0/img_000000013_Default1_000.tif]

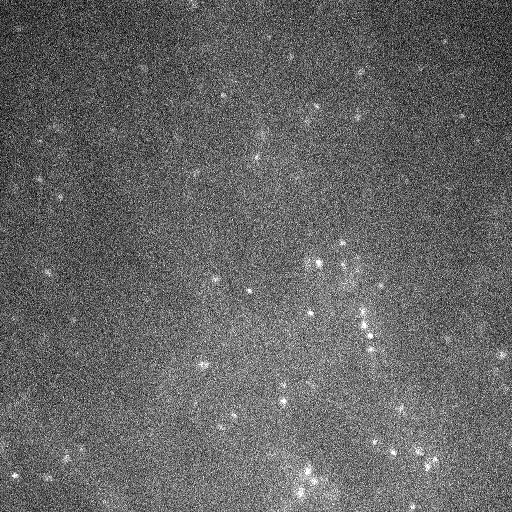

Supplement: Source code 1. [file elife-50629-code1.zip › PAmCherry-PBP2/slow/samplemovie/Pos0/img_000000008_Default1_000.tif]

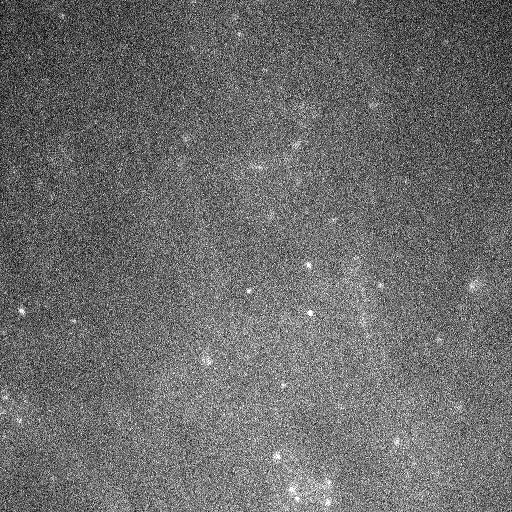

Supplement: Source code 1. [file elife-50629-code1.zip › PAmCherry-PBP2/slow/samplemovie/Pos0/img_000000017_Default1_000.tif]

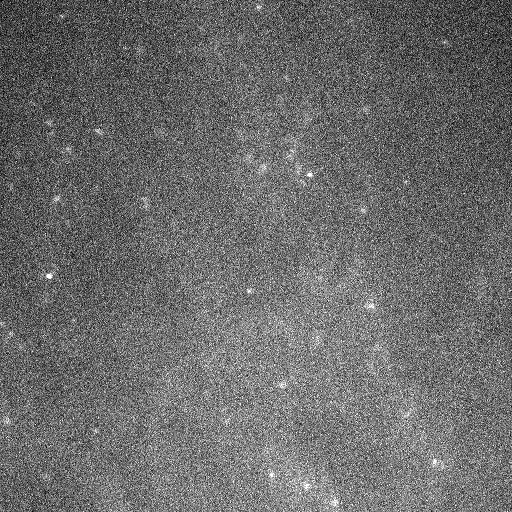

Supplement: Source code 1. [file elife-50629-code1.zip › PAmCherry-PBP2/slow/samplemovie/Pos0/img_000000029_Default1_000.tif]

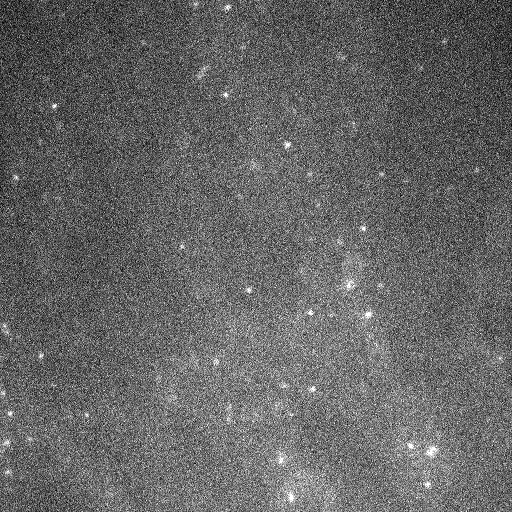

Supplement: Source code 1. [file elife-50629-code1.zip › PAmCherry-PBP2/slow/samplemovie/Pos0/img_000000003_Default1_000.tif]

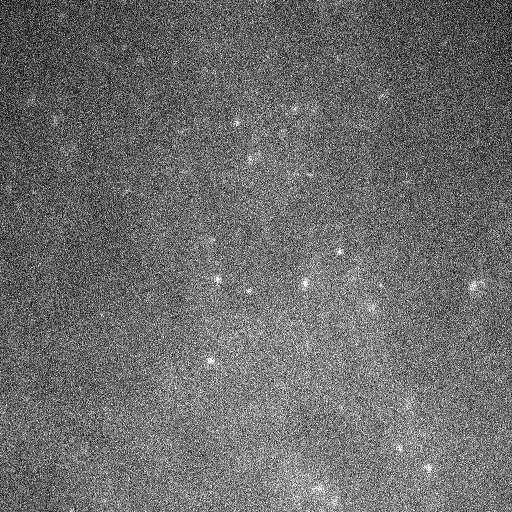

Supplement: Source code 1. [file elife-50629-code1.zip › PAmCherry-PBP2/slow/samplemovie/Pos0/img_000000022_Default1_000.tif]

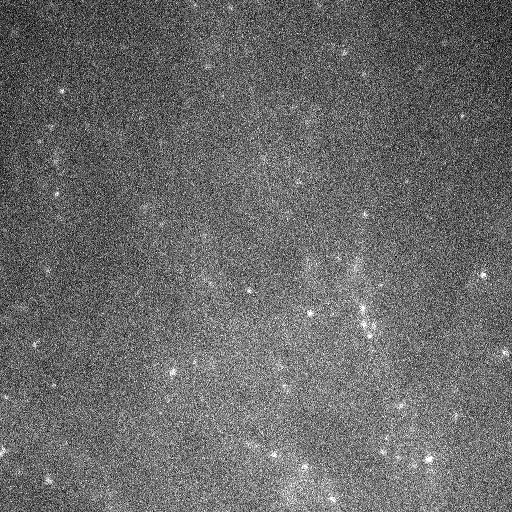

Supplement: Source code 1. [file elife-50629-code1.zip › PAmCherry-PBP2/slow/samplemovie/Pos0/img_000000010_Default1_000.tif]

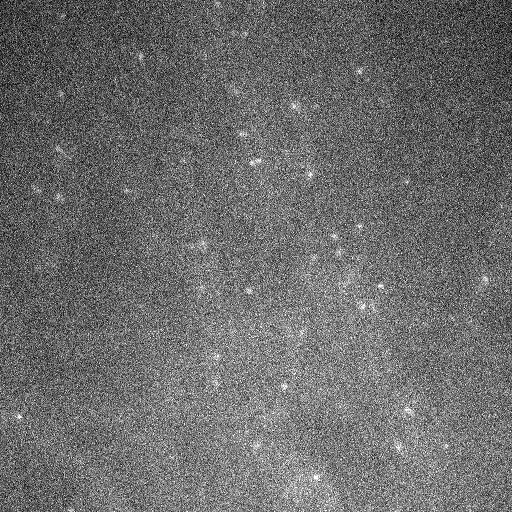

Supplement: Source code 1. [file elife-50629-code1.zip › PAmCherry-PBP2/slow/samplemovie/Pos0/img_000000025_Default1_000.tif]

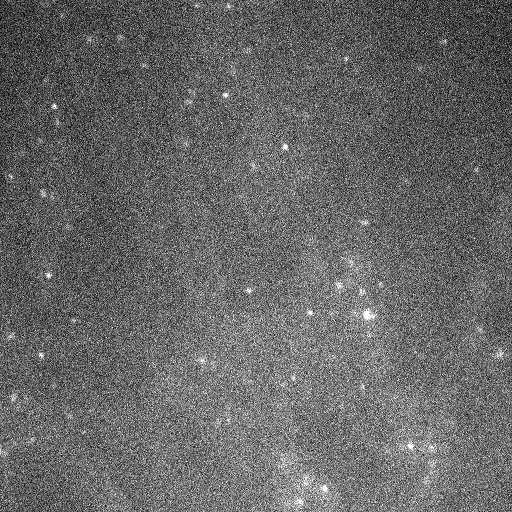

Supplement: Source code 1. [file elife-50629-code1.zip › PAmCherry-PBP2/slow/samplemovie/Pos0/img_000000004_Default1_000.tif]

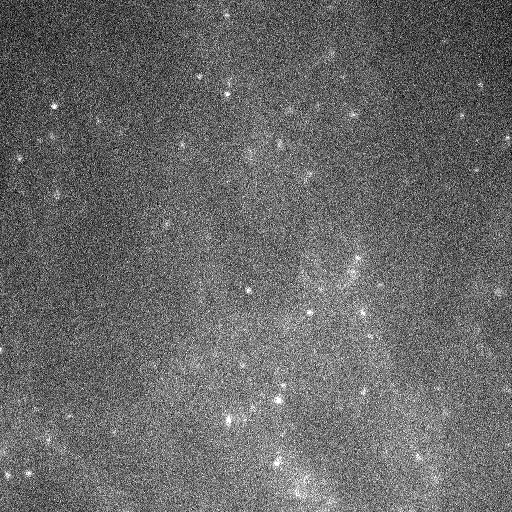

Supplement: Source code 1. [file elife-50629-code1.zip › PAmCherry-PBP2/slow/samplemovie/Pos0/img_000000000_Default1_000.tif]

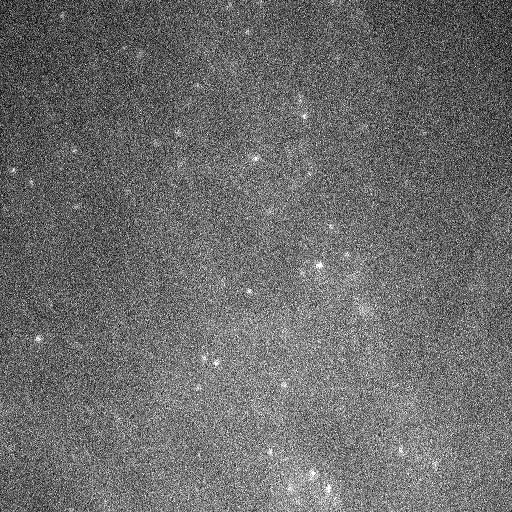

Supplement: Source code 1. [file elife-50629-code1.zip › PAmCherry-PBP2/slow/samplemovie/Pos0/img_000000021_Default1_000.tif]

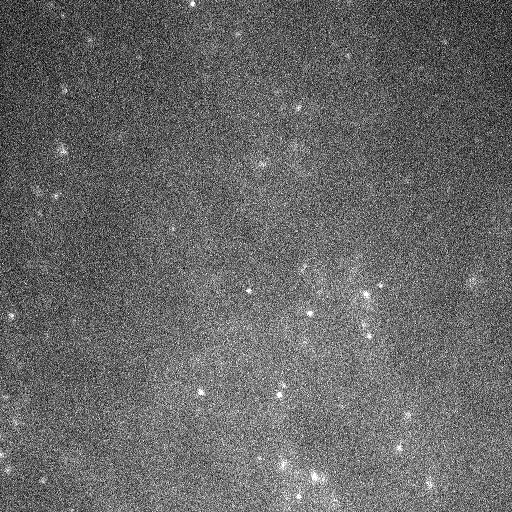

Supplement: Source code 1. [file elife-50629-code1.zip › PAmCherry-PBP2/slow/samplemovie/Pos0/img_000000014_Default1_000.tif]

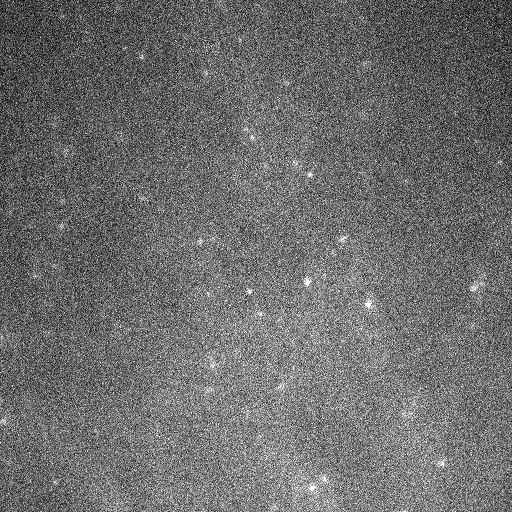

Supplement: Source code 1. [file elife-50629-code1.zip › PAmCherry-PBP2/slow/samplemovie/Pos0/img_000000030_Default1_000.tif]

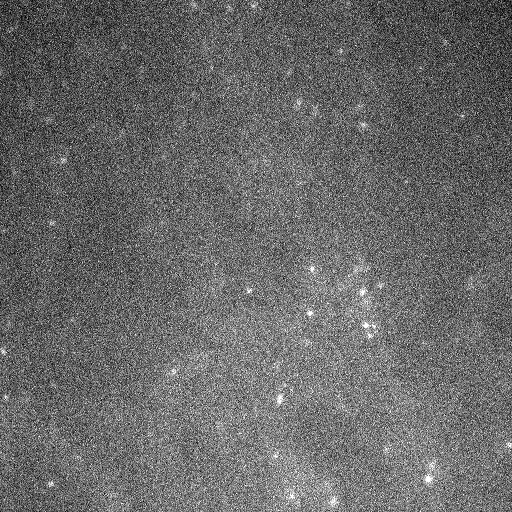

Supplement: Source code 1. [file elife-50629-code1.zip › PAmCherry-PBP2/slow/samplemovie/Pos0/img_000000011_Default1_000.tif]

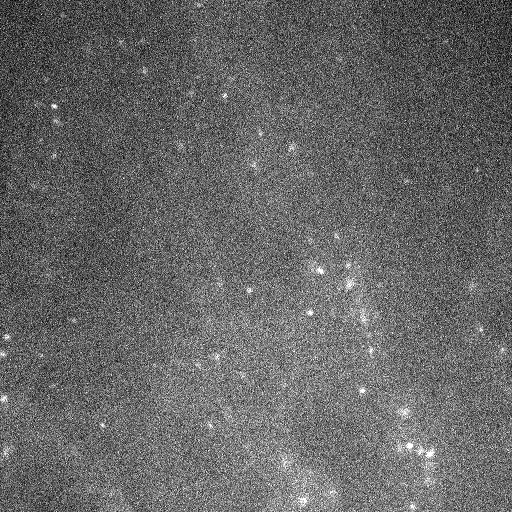

Supplement: Source code 1. [file elife-50629-code1.zip › PAmCherry-PBP2/slow/samplemovie/Pos0/img_000000005_Default1_000.tif]

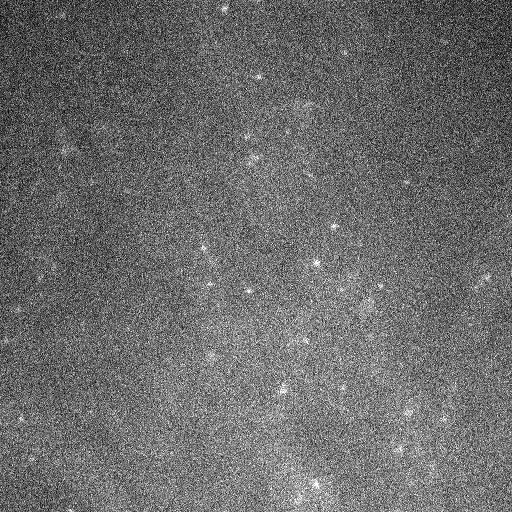

Supplement: Source code 1. [file elife-50629-code1.zip › PAmCherry-PBP2/slow/samplemovie/Pos0/img_000000024_Default1_000.tif]

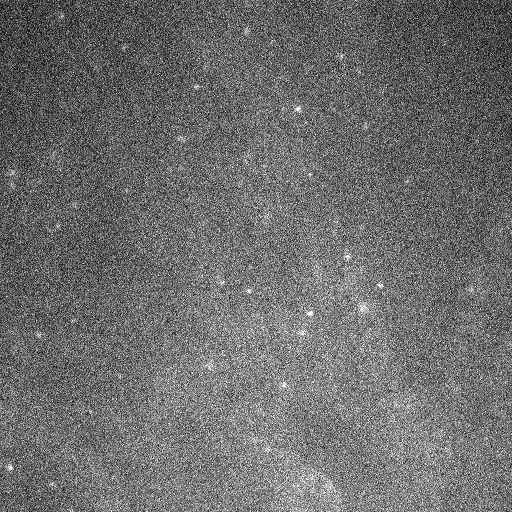

Supplement: Source code 1. [file elife-50629-code1.zip › PAmCherry-PBP2/slow/samplemovie/Pos0/img_000000020_Default1_000.tif]

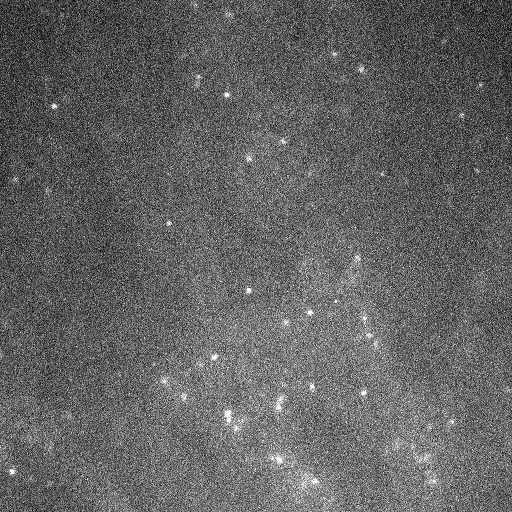

Supplement: Source code 1. [file elife-50629-code1.zip › PAmCherry-PBP2/slow/samplemovie/Pos0/img_000000001_Default1_000.tif]

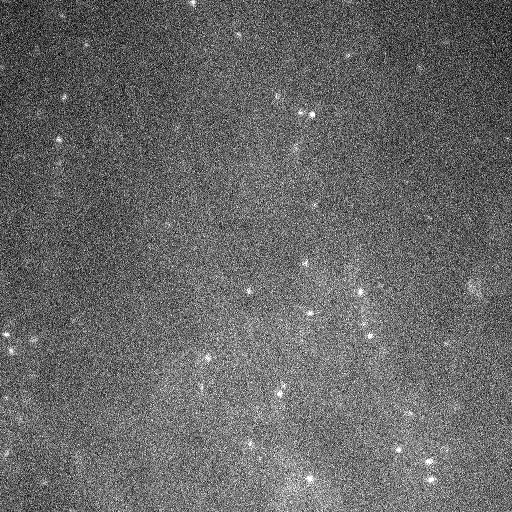

Supplement: Source code 1. [file elife-50629-code1.zip › PAmCherry-PBP2/slow/samplemovie/Pos0/img_000000015_Default1_000.tif]

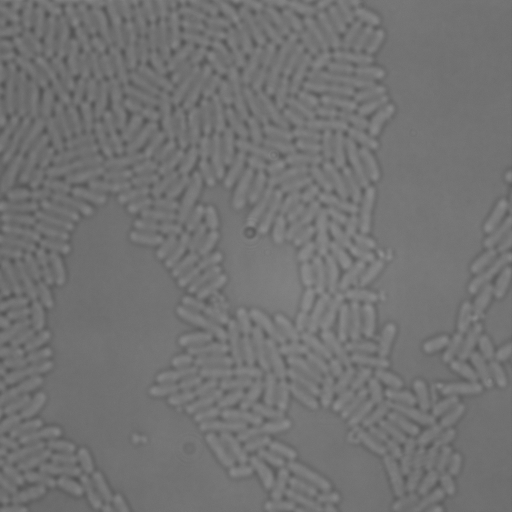

Supplement: Source code 1. [file elife-50629-code1.zip › PAmCherry-PBP2/slow/samplemovie/AVG_Pos1.tif]

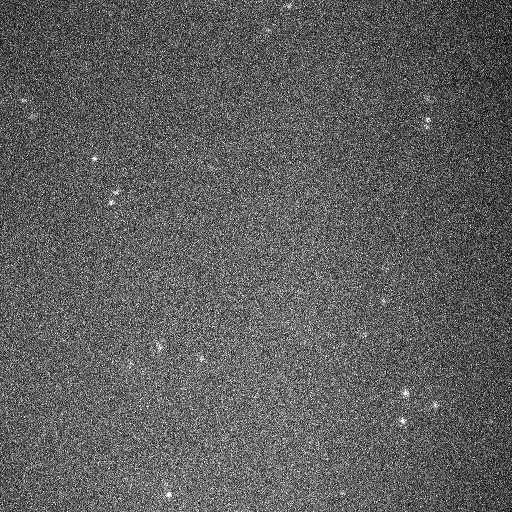

Supplement: Source code 1. [file elife-50629-code1.zip › PAmCherry-PBP2/fast/samplemovie/Pos0/img_000000023_Default0_000.tif]

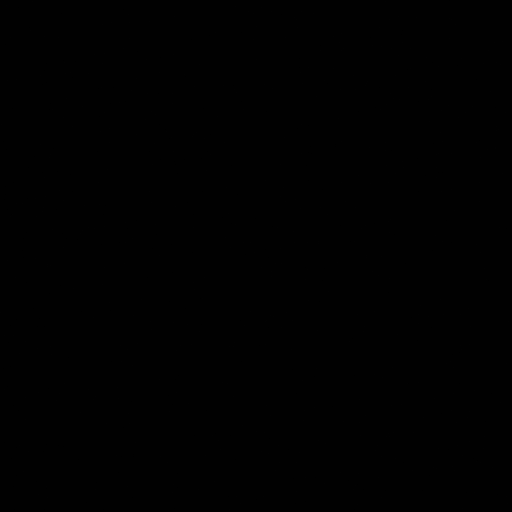

Supplement: Source code 1. [file elife-50629-code1.zip › PAmCherry-PBP2/fast/samplemovie/Pos0/img_000000002_Default0_000.tif]

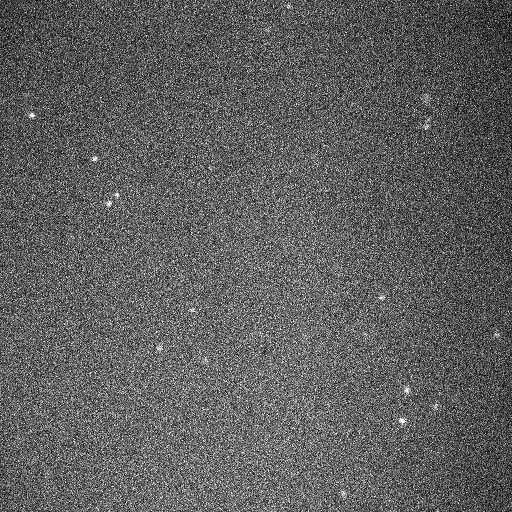

Supplement: Source code 1. [file elife-50629-code1.zip › PAmCherry-PBP2/fast/samplemovie/Pos0/img_000000028_Default0_000.tif]

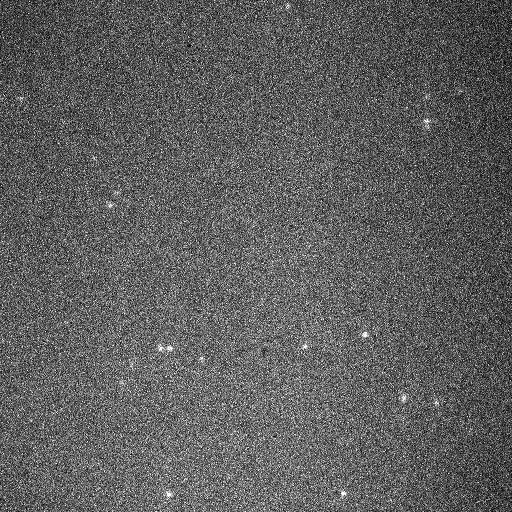

Supplement: Source code 1. [file elife-50629-code1.zip › PAmCherry-PBP2/fast/samplemovie/Pos0/img_000000016_Default0_000.tif]

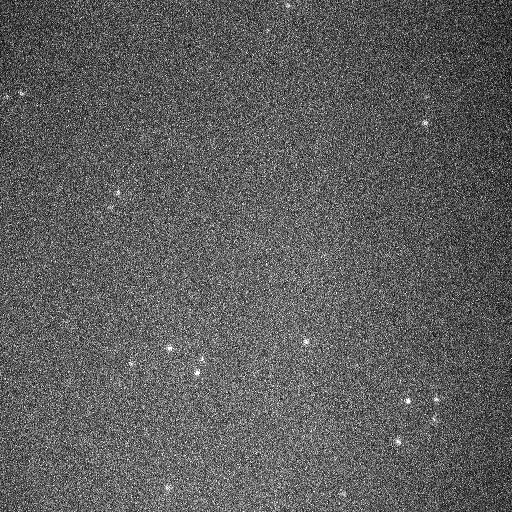

Supplement: Source code 1. [file elife-50629-code1.zip › PAmCherry-PBP2/fast/samplemovie/Pos0/img_000000009_Default0_000.tif]

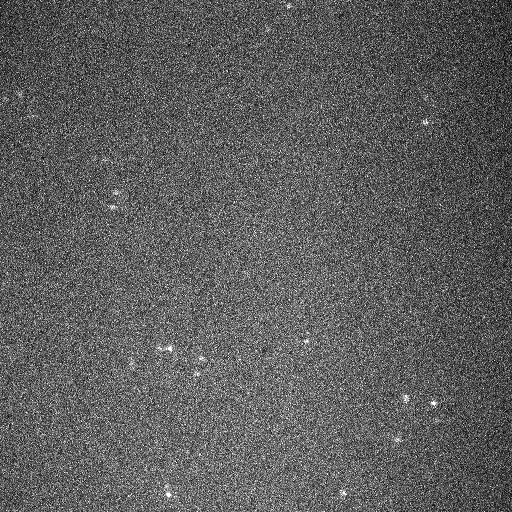

Supplement: Source code 1. [file elife-50629-code1.zip › PAmCherry-PBP2/fast/samplemovie/Pos0/img_000000012_Default0_000.tif]

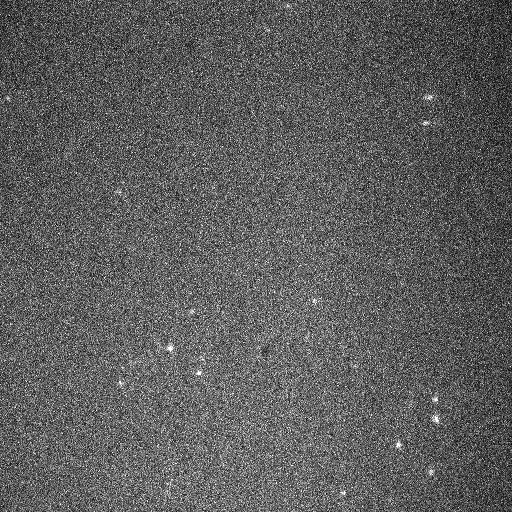

Supplement: Source code 1. [file elife-50629-code1.zip › PAmCherry-PBP2/fast/samplemovie/Pos0/img_000000006_Default0_000.tif]

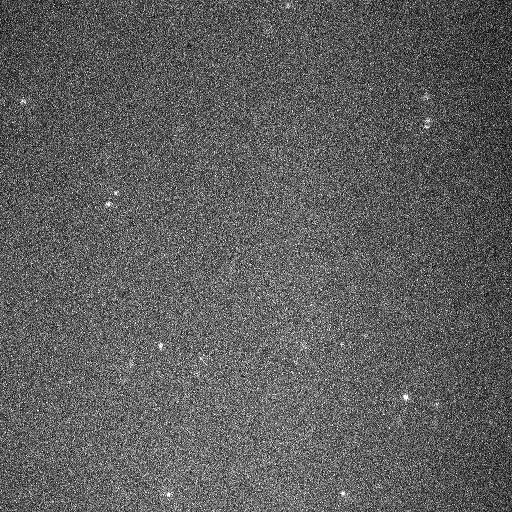

Supplement: Source code 1. [file elife-50629-code1.zip › PAmCherry-PBP2/fast/samplemovie/Pos0/img_000000019_Default0_000.tif]

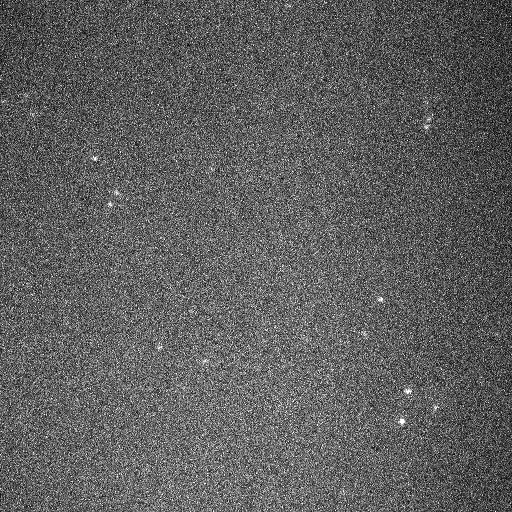

Supplement: Source code 1. [file elife-50629-code1.zip › PAmCherry-PBP2/fast/samplemovie/Pos0/img_000000027_Default0_000.tif]

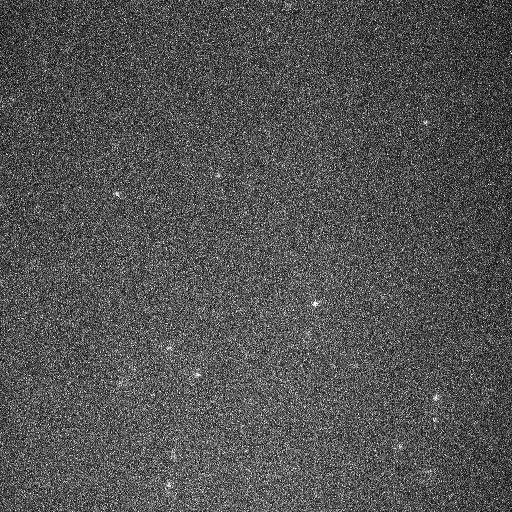

Supplement: Source code 1. [file elife-50629-code1.zip › PAmCherry-PBP2/fast/samplemovie/Pos0/img_000000003_Default0_000.tif]

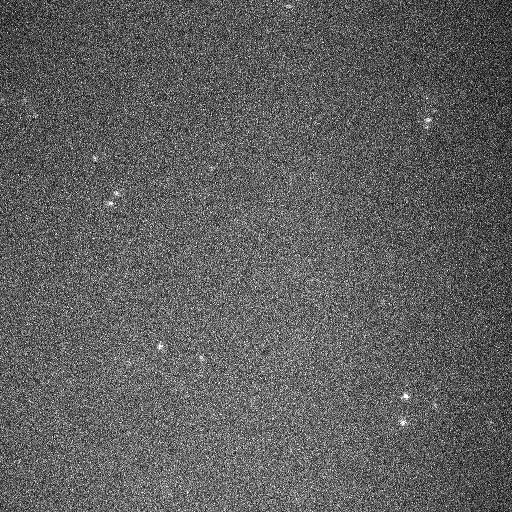

Supplement: Source code 1. [file elife-50629-code1.zip › PAmCherry-PBP2/fast/samplemovie/Pos0/img_000000022_Default0_000.tif]

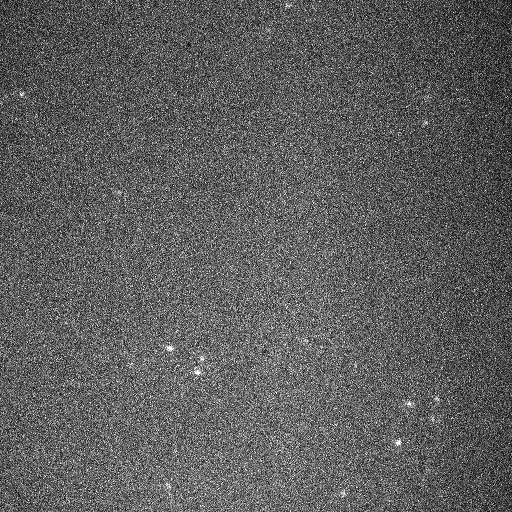

Supplement: Source code 1. [file elife-50629-code1.zip › PAmCherry-PBP2/fast/samplemovie/Pos0/img_000000008_Default0_000.tif]

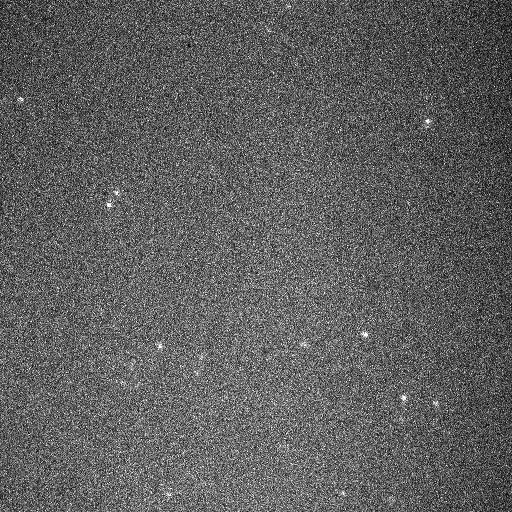

Supplement: Source code 1. [file elife-50629-code1.zip › PAmCherry-PBP2/fast/samplemovie/Pos0/img_000000017_Default0_000.tif]

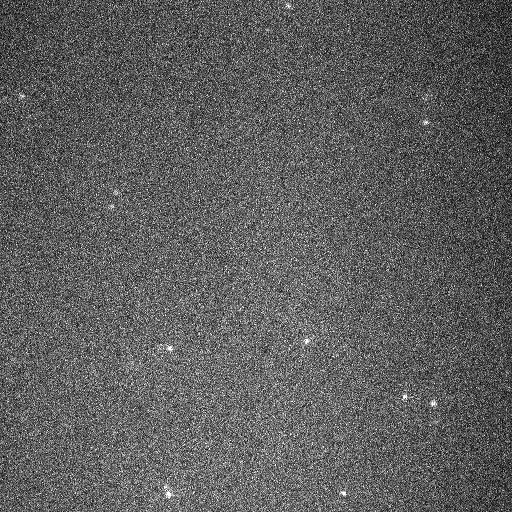

Supplement: Source code 1. [file elife-50629-code1.zip › PAmCherry-PBP2/fast/samplemovie/Pos0/img_000000013_Default0_000.tif]

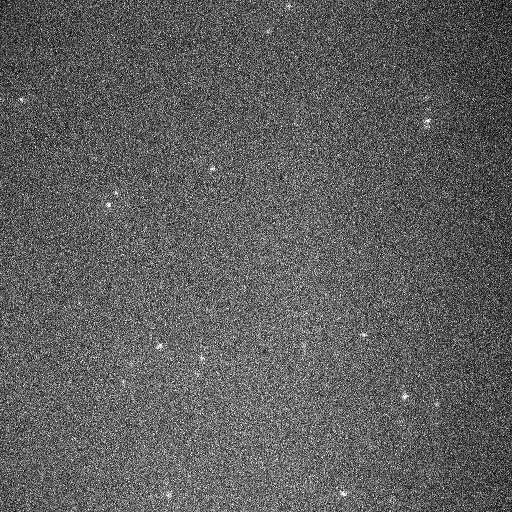

Supplement: Source code 1. [file elife-50629-code1.zip › PAmCherry-PBP2/fast/samplemovie/Pos0/img_000000018_Default0_000.tif]

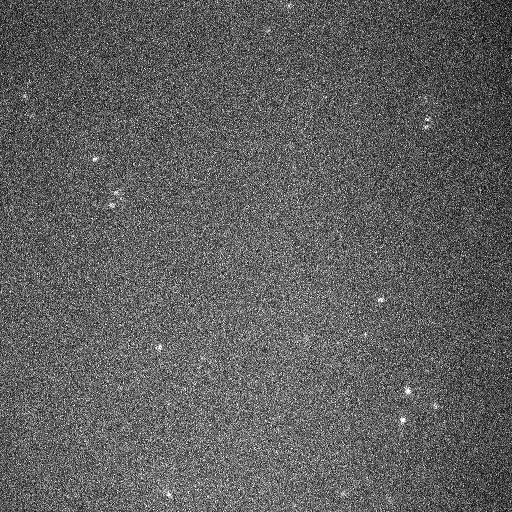

Supplement: Source code 1. [file elife-50629-code1.zip › PAmCherry-PBP2/fast/samplemovie/Pos0/img_000000026_Default0_000.tif]

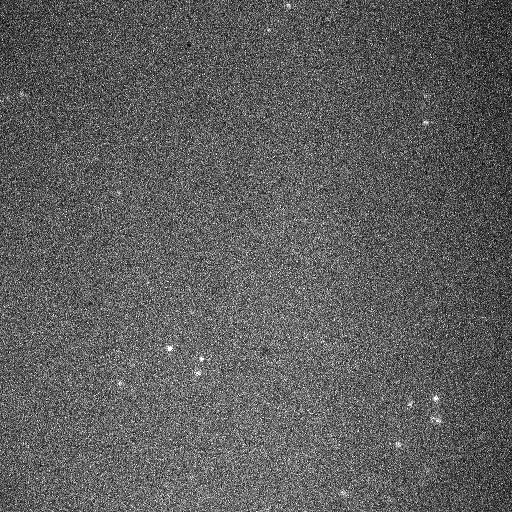

Supplement: Source code 1. [file elife-50629-code1.zip › PAmCherry-PBP2/fast/samplemovie/Pos0/img_000000007_Default0_000.tif]

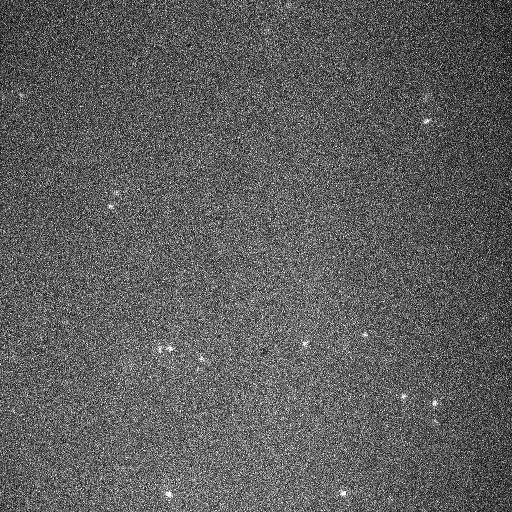

Supplement: Source code 1. [file elife-50629-code1.zip › PAmCherry-PBP2/fast/samplemovie/Pos0/img_000000014_Default0_000.tif]

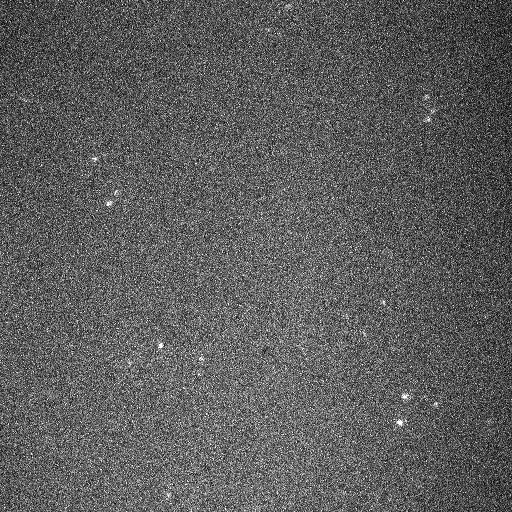

Supplement: Source code 1. [file elife-50629-code1.zip › PAmCherry-PBP2/fast/samplemovie/Pos0/img_000000021_Default0_000.tif]

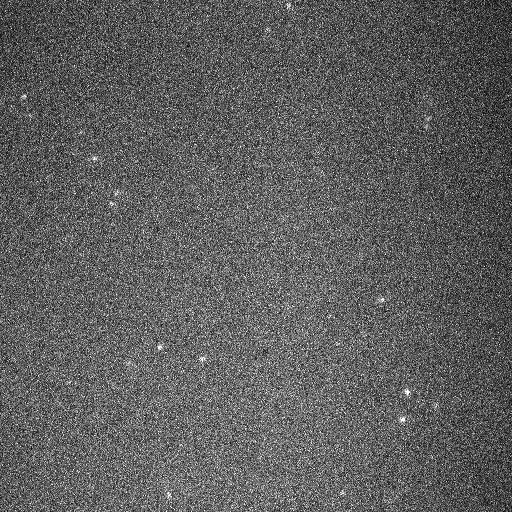

Supplement: Source code 1. [file elife-50629-code1.zip › PAmCherry-PBP2/fast/samplemovie/Pos0/img_000000025_Default0_000.tif]

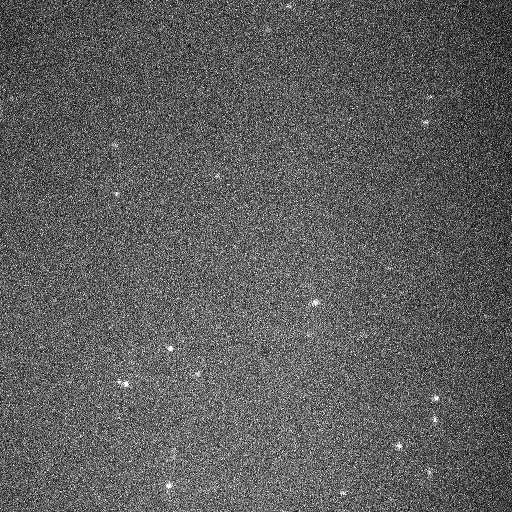

Supplement: Source code 1. [file elife-50629-code1.zip › PAmCherry-PBP2/fast/samplemovie/Pos0/img_000000004_Default0_000.tif]

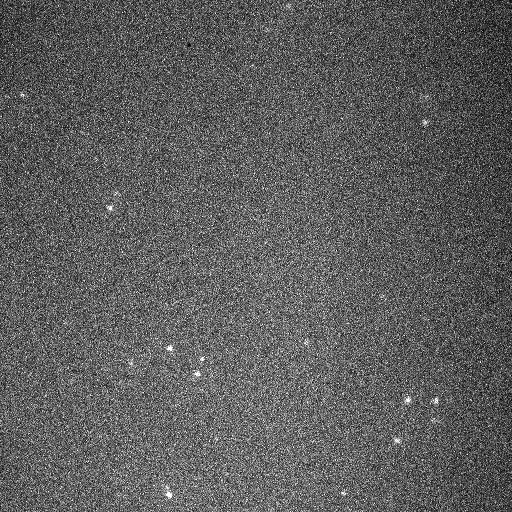

Supplement: Source code 1. [file elife-50629-code1.zip › PAmCherry-PBP2/fast/samplemovie/Pos0/img_000000010_Default0_000.tif]

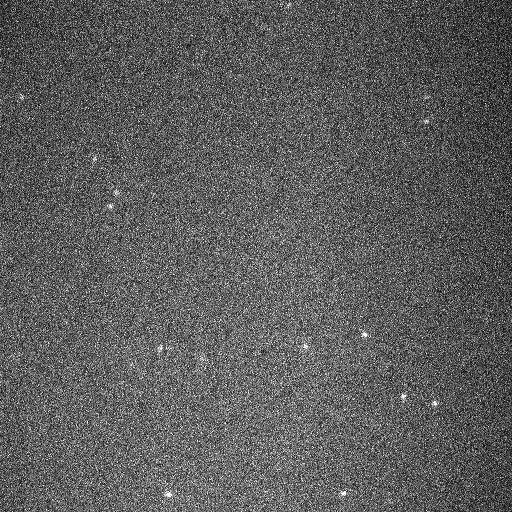

Supplement: Source code 1. [file elife-50629-code1.zip › PAmCherry-PBP2/fast/samplemovie/Pos0/img_000000015_Default0_000.tif]

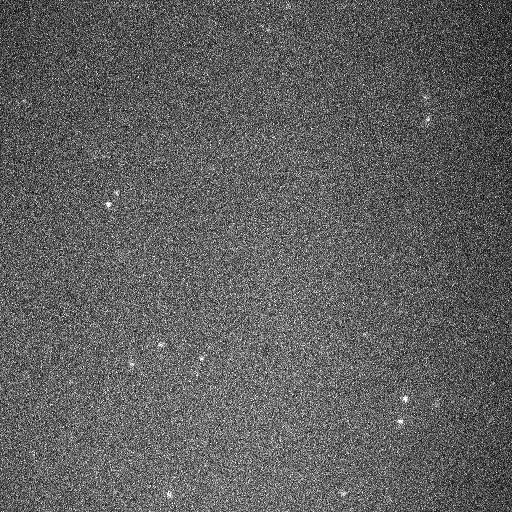

Supplement: Source code 1. [file elife-50629-code1.zip › PAmCherry-PBP2/fast/samplemovie/Pos0/img_000000020_Default0_000.tif]

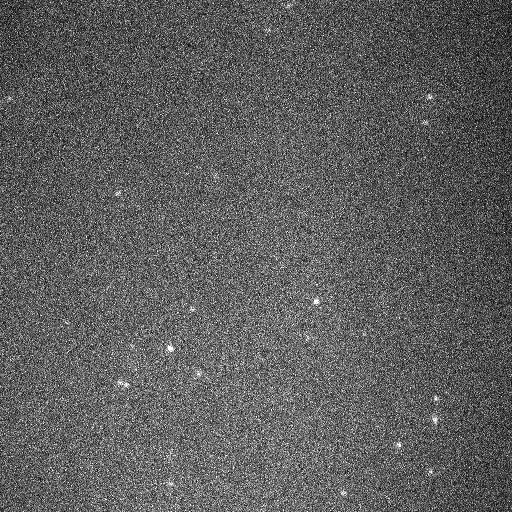

Supplement: Source code 1. [file elife-50629-code1.zip › PAmCherry-PBP2/fast/samplemovie/Pos0/img_000000005_Default0_000.tif]

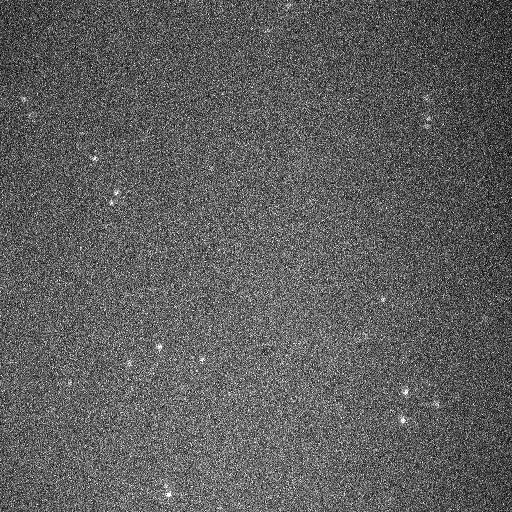

Supplement: Source code 1. [file elife-50629-code1.zip › PAmCherry-PBP2/fast/samplemovie/Pos0/img_000000024_Default0_000.tif]

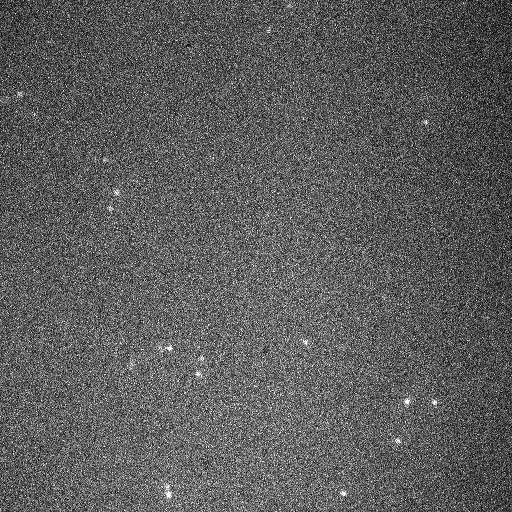

Supplement: Source code 1. [file elife-50629-code1.zip › PAmCherry-PBP2/fast/samplemovie/Pos0/img_000000011_Default0_000.tif]

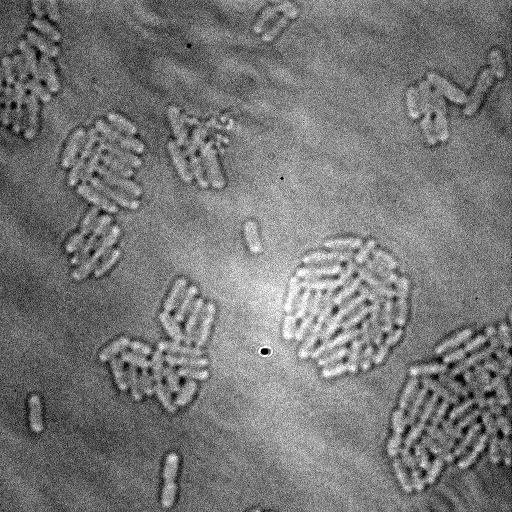

Supplement: Source code 1. [file elife-50629-code1.zip › PAmCherry-PBP2/fast/samplemovie/AVG_Pos1.tif]
